# Comparison of Transnasal Humidified Rapid-Insufflation Ventilatory Exchange (THRIVE) and Conventional Facemask Ventilation During Electroconvulsive Therapy in Major Mental Disorders: A Preliminary Open-Label Randomized Controlled Trial

**DOI:** 10.31083/AP39942

**Published:** 2026-01-08

**Authors:** Can-Jin Deng, Sha Nie, Rong Zeng, Jian-Xin Mai, Xiong Huang, Shi-Chao Xu, Xin-Hu Yang, Qing-Bin Zeng, Xing-Bing Huang, Wei Zheng

**Affiliations:** ^1^Department of Psychiatry, The Affiliated Brain Hospital, Guangzhou Medical University, 510370 Guangzhou, Guangdong, China; ^2^Key Laboratory of Neurogenetics and Channelopathies of Guangdong Province and the Ministry of Education of China, Guangzhou Medical University, 510370 Guangzhou, Guangdong, China; ^3^Department of Psychiatry, The Third People’s Hospital of Foshan, 528041 Foshan, Guangdong, China

**Keywords:** electroconvulsive therapy, anesthesia, transnasal humidified rapid-insufflation ventilatory exchange, major mental disorders

## Abstract

**Background:**

Transnasal Humidified Rapid-Insufflation Ventilatory Exchange (THRIVE) technique prolongs apnea duration. However, there is limited knowledge regarding the effectiveness and safety of THRIVE technique compared to conventional facemask ventilation during electroconvulsive therapy (ECT) in Chinese patients with major mental disorders.

**Methods:**

Seventy adult individuals with major mental disorders (schizophrenia, n = 17; bipolar disorder, n = 25; major depressive disorder, n = 28) undergoing their first ECT session were assigned to either the THRIVE group (n = 35) or the facemask group (n = 35) based on the random sequence. The primary outcome was the lowest peripheral oxygen saturation (SpO_2_) levels. Secondary outcomes included the incidence of oxygen desaturation, electroencephalogram seizure duration, stimulation dosage, mean arterial pressure (MAP), average SpO_2_ levels, and heart rate (HR). Airway-related complications were documented within 24 hours following ECT.

**Results:**

In the THRIVE group, the lowest SpO_2_ levels were notably higher than those in the facemask group (*p* < 0.05). Patients receiving THRIVE technique had consistently higher average SpO_2_ levels than those receiving conventional facemask ventilation (*p* < 0.05). The incidence of oxygen desaturation in THRIVE group was lower than that in facemask group (*p* > 0.05). Moreover, significant differences between two study groups were not observed in terms of electroencephalogram seizure duration, stimulation dosage, MAP, and HR (all *ps* > 0.05). No airway-related complications were reported in either group.

**Conclusions:**

In this preliminary open-label randomized controlled trial, the THRIVE technique appeared to be more effective than conventional facemask ventilation in preserving SpO_2_ levels during ECT in major mental disorders, establishing itself as a safe and effective oxygenation alternative.

**Trial Registration:**

No: ChiCTR2400084318, https://www.chictr.org.cn/showproj.html?proj=229304.

## Main Points

(1) The Transnasal Humidified Rapid-Insufflation Ventilatory Exchange (THRIVE) technique is a novel intraoperative ventilatory technique.

(2) This is the first study to examine the efficacy and safety of the THRIVE technique compared to conventional facemask ventilation in Chinese patients with major mental disorders during electroconvulsive therapy (ECT) procedure.

(3) The THRIVE technique demonstrates superiority over conventional facemask ventilation in preserving peripheral oxygen saturation levels during the ECT procedure.

## 1. Introduction

Electroconvulsive therapy (ECT) is a noninvasive brain stimulation (NIBS) technique. It has been considered an effective physical treatment for a broad spectrum of severe mental disorders [1, 2, 3]. To prevent injuries resulting from widespread epileptic seizures during the ECT procedure, patients must undergo general anesthesia [4, 5]. Conventional facemask ventilation has conventionally been the primary technique for airway management during ECT procedures; however, it may not be the optimal choice [6, 7]. When conventional facemask ventilation is used in ECT procedures, the incidence of oxygen desaturation could range from 2.5% to 29.0% [8, 9, 10, 11, 12]. Oxygen desaturation during ECT could shorten the seizure duration and even result in arrhythmias or myocardial infarction [13, 14]. Moreover, conventional facemask ventilation increases the risk of aspiration of gastric contents [15, 16]; it might be challenging for patients with facial tumors, large jaws, thick beards, or edentulous conditions [17, 18, 19]. Therefore, there is a need for the development of advanced airway devices and technologies.

Transnasal Humidified Rapid-Insufflation Ventilatory Exchange (THRIVE), a novel ventilation technique, involves supplying patients with warm, humidified, high-flow oxygen to prolong apneic oxygenation under general anesthesia [20, 21]. The THRIVE technique removes carbon dioxide (CO_2_) more efficiently than conventional apneic oxygenation, extending the safe apnea time [22, 23]. Previous studies have reported that safe apnea time maintained with the THRIVE technique ranges from 20 minutes to 65 minutes under general anesthesia [22, 24, 25]. When the THRIVE technique is used during the induction of anesthesia, it does not further increase the risk of gastric insufflation [26, 27]. The THRIVE technique is hands-free, allowing anesthesiologists to devote more attention to patient care. The method has found applications in various settings, such as laryngotracheal surgery [22, 28], rapid sequence intubation (RSI) [21, 29], and endoscopy [30, 31].

The safety and effectiveness of using the THRIVE technique during ECT procedures have been demonstrated by several studies [27, 32, 33, 34], although with inconsistent findings. For example, a non-randomized cross-over study involving 201 adult participants with a body mass index (BMI) under 40 kg/m^2^ found a 0.5% incidence of oxygen desaturation during ECT with the THRIVE technique [32]. However, Zhu *et al*. [27] reported a 5.3% incidence of desaturation during ECT with the THRIVE technique in 150 adult patients with a BMI lower than 24 kg/m^2^. In most studies, the peripheral oxygen saturation (SpO_2_) levels of all patients undergoing the THRIVE technique during ECT procedure remained > 95% [33, 34].

In this preliminary open-label randomized controlled trial (RCT), participants with major mental disorders (i.e., major depressive disorder (MDD), bipolar disorder (BD), and schizophrenia) were divided into the THRIVE and facemask groups during ECT procedures. This study primarily aimed to compare the efficacy (i.e., SpO_2_ level ≥ 92%) and safety (e.g., the rate of airway-related complications) of the THRIVE technique with conventional facemask ventilation throughout ECT procedures. Drawing upon the findings of previous studies [27, 32, 33, 34], we hypothesized that the THRIVE technique could be used safely and effectively in adult individuals with major mental disorders during ECT procedures. Furthermore, we anticipated that the THRIVE technique would outperform conventional facemask ventilation in preserving SpO_2_ levels during ECT procedures.

## 2. Methods

### 2.1 Study Design and Participants

This study was a preliminary open-label RCT, which was carried out at the Affiliated Brain Hospital, Guangzhou Medical University, from 01 June 2024 until 01 August 2024. The Institutional Review Board (IRB) of the Affiliated Brain Hospital, Guangzhou Medical University, approved this trial (approval number: 2024034). The protocol was registered in the Clinical Trials Registry, China (registered number: ChiCTR2400084318) on May 14, 2024. The reporting of this study complied with the Consolidated Standards of Reporting Trials (CONSORT) guidelines [35]. All participants provided informed written consent. This study was conducted in compliance with the Declaration of Helsinki and national ethical regulations.

Participants were recruited from the inpatient unit of the Affiliated Brain Hospital, Guangzhou Medical University, a 1800-bed psychiatric treatment center located in Guangzhou, China. The eligibility criteria for this study included: (1) adult patients (aged 18–65 years) who had been diagnosed with schizophrenia, BD, or MDD according to the Diagnostic and Statistical Manual of Mental Disorders, 5th edition (DSM-V); (2) patients with a BMI ranging from 18.5 to 23.9 kg/m^2^ [27]; (3) patients with American Society of Anesthesiologists (ASA) physical status I-II [36]; and (4) patients who were able to receive THRIVE technique following instructions properly.

The criteria for exclusion in this study included: (1) the presence of other psychiatric disorders as defined by DSM-V, such as alcohol or substance use disorder; (2) those suffering from obstructive sleep apnea syndrome (OSAS) along with nasopharyngeal abnormalities, epistaxis, or a history of nasal surgery; (3) known or anticipated airway obstruction requiring intubation; (4) patients suffering from severe or unstable somatic conditions (e.g., emphysema and chronic obstructive pulmonary disease); (5) pregnant or lactating individuals; and (6) contraindications to ECT or anesthesia.

Participants were recruited only for their first ECT session, during which ECT was administered using either the THRIVE technique or conventional facemask ventilation.

### 2.2 Sample Size and Randomization

The sample size of 70 in this study was established according to a prior RCT (n = 70) that applied THRIVE for painless endoscopic mucosal resection of colorectal polyps [37]. The Statistical Package for the Social Sciences (SPSS) software (version 23.0, IBM SPSS Statistics for Windows, Armonk, NY, USA) was employed to generate the random sequence with a 1:1 ratio. A researcher, independent of the assessment and treatment processes, assigned patients to either the THRIVE group or the facemask group based on the random sequence. Due to the different appearance of the masks and high-flow nasal cannulas, it is not feasible for patients and assessors to be blinded.

### 2.3 ECT Procedure

Adult patients undergoing ECT received bilateral electrode placement using the Thymatron System IV device (Somatics LLC, Lake Bluff, IL, USA). The seizure threshold determination was based on the half-age dosing method, with the energy percentage calculated as the age multiplied by 0.5 [38]. After pre-oxygenation, all patients underwent general anesthesia following a standardized anesthesia protocol. Premedication anesthesia was induced by intravenous injection of atropine (0.5 mg) and propofol (1.5–2.0 mg/kg) before ECT administration. Once the patient became unconscious, intravenous succinylcholine (0.8–1.0 mg/kg) was administered to relax the muscles.

#### 2.3.1 THRIVE Group 

The Optiflow^®^ THRIVE (Fisher & Paykel Healthcare, Auckland, New Zealand) is a humidifier with an integrated flow generator that delivers humidified, warmed, and high-flow oxygen at up to 70 L/min. Before using the THRIVE device, the humidifier must be heated for five minutes to reach a temperature of 37 °C [39]. Patients were fitted with appropriate high-flow nasal cannulas. During the 3-minute preoxygenation phase, 100% oxygen was administered at a flow rate of 30 L/min [36]. Exhalation was performed through the mouth, while inhalation was through the nose [33]. A flow rate of 70 L/min was maintained during apnea, even after administering muscle relaxants [33]. After sufficient spontaneous breathing, the flow rate was gradually reduced to 30 L/min and sustained at that level until the patient fully regained consciousness.

#### 2.3.2 Facemask Group 

In the facemask group, preoxygenation with 100% oxygen was carried out for 3 minutes at a flow rate of 10 L/min, with no positive airway pressure applied, using an appropriately sized mask [21]. Immediately following the muscle relaxant injection, patients were ventilated using conventional facemasks and 100% oxygen until they fully regained consciousness. Until patients resumed spontaneous breathing, they received 100% oxygen using a mask, with a flow rate of 10 L/min.

### 2.4 Primary and Secondary Outcomes

All data were collected during the patients’ first ECT session. During the ECT procedure, specific time points were defined, including the baseline (T_0_), induction of anesthesia (T_1_), delivery of ECT stimulus (T_2_), recovery of spontaneous breathing (T_3_), and regaining consciousness (T_4_). At these designated time points (T_0_–T_4_), mean arterial pressure (MAP), heart rate (HR), and SpO_2_ levels were recorded.

The lowest SpO_2_ levels were continuously measured throughout the ECT procedure using a pulse oximeter as the primary outcome [27, 33]. Secondary outcomes encompassed the incidence of oxygen desaturation (defined as a SpO_2_ level < 92%), electroencephalogram (EEG) seizure duration, stimulation dosage, and vital signs (including average SpO_2_ levels, HR, and MAP) at the predefined time points. The time intervals from T_1_ to T_3_ and T_4_ were measured, determining the apnea duration and consciousness recovery time, respectively [40].

Airway-related complications, such as nose bleeds, nasal dryness, pain, and itching, were recorded for up to 24 hours following the ECT procedure.

### 2.5 Statistical Analysis

All statistical analyses were conducted using the SPSS software (version 23.0, IBM Corp., Armonk, NY, USA). The normal distribution of quantitative data was assessed via the Shapiro–Wilk test. Quantitative data were properly presented as either means and standard deviations (SD) or medians and interquartile ranges (IQR), while qualitative data were described using frequency (N) and percentage (%). Categorical variables were compared using either Pearson’s chi-square test or Fisher’s exact test, while continuous variables were assessed through either the Mann–Whitney U test or the independent samples *t*-test, as appropriate. Linear mixed models with a Bonferroni correction were utilized to compare HR, MAP, and SpO_2_ levels across multiple time points between the THRIVE and the facemask groups. Statistical significance was set at *p* < 0.05.

## 3. Results

### 3.1 Demographic and Clinical Features of Patients

**Fig. 1.**
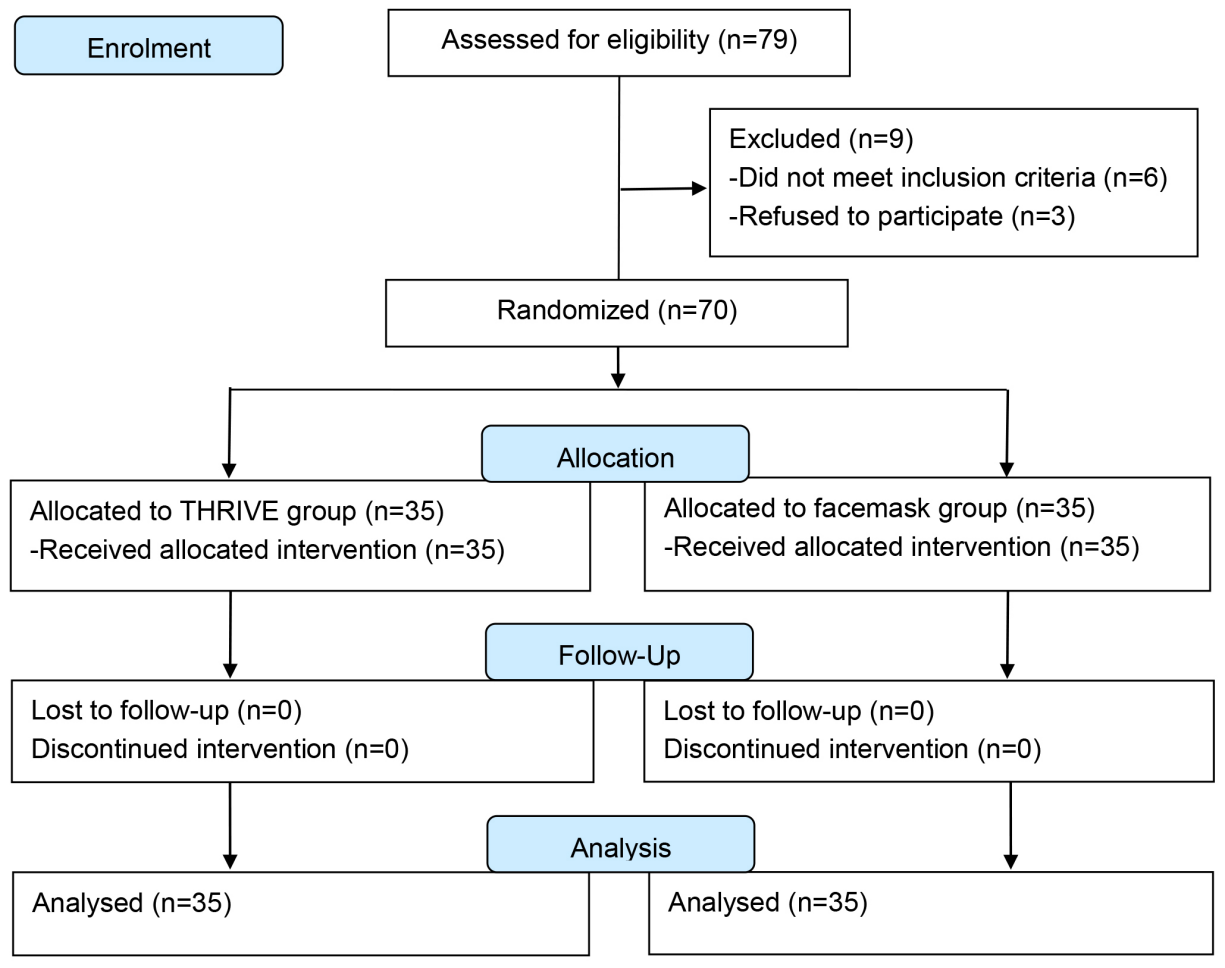
**CONSORT flowchart of recruitment**. CONSORT, Consolidated Standards of Reporting Trials; THRIVE, Transnasal Humidified Rapid-Insufflation Ventilatory Exchange.

The CONSORT flowchart detailing the recruitment process is presented in Fig. 1. A total of 79 patients were assessed for eligibility. Of these, nine were excluded (three refusals to participate and six non-fulfillments of inclusion criteria). Finally, 70 patients with major mental disorders (schizophrenia, n = 17; BD, n = 25; MDD, n = 28) met the enrollment criteria for this study. Table 1 summarizes the clinical characteristics and demographics of the 70 patients who underwent ECT, with 35 receiving conventional facemask ventilation and 35 receiving the THRIVE technique. In terms of sex, weight, hemoglobin levels, age, baseline SpO_2_ levels, BMI, diagnosis, ASA grade, and education levels, there were no significant differences between the THRIVE and facemask groups (all *ps* > 0.05).

**Table 1.** **Comparison of baseline characteristics between THRIVE and facemask groups**.

Variables		THRIVE group (n = 35)		Facemask group (n = 35)		Statistics		
		Medians	IQR	Medians	IQR	*z*	df	*p*
Age (years)		27.0	23.0, 39.0	28.0	22.0, 35.0	0.11	—^a^	0.916
Education (years)		13.0	9.0, 15.0	15.0	9.0, 16.0	–0.98	—^a^	0.329
Weight (kg)		60.0	55.0, 67.0	54.0	51.0, 64.0	1.64	—^a^	0.101
Baseline SpO_2_ (%)		98.0	97.0, 99.0	98.0	97.0, 98.0	0.98	—^a^	0.328
Variables		THRIVE group (n = 35)		Facemask group (n = 35)		Statistics		
		Mean	SD	Mean	SD	*t*	df	*p*
BMI (kg/m^2^)		21.2	1.7	20.9	1.7	–0.79	68	0.433
Hemoglobin (g/L)		135.4	15.9	129.7	13.9	–1.59	68	0.116
Variables		THRIVE group (n = 35)		Facemask group (n = 35)		Statistics		
		N	%	N	%	$\chi^{2}$	df	*p*
Male		22	62.9	15	42.9	2.81	1	0.094
Diagnosis						1.63	2	0.443
	Schizophrenia	8	22.9	9	25.7			
	BD	15	42.9	10	28.6			
	MDD	12	34.3	16	45.7			
ASA grade						0.08	1	0.780
	I	26	74.3	27	77.1			
	II	9	25.7	8	22.9			

^a^Mann–Whitney U test. ASA, American Society of Anesthesiologists; BMI, body mass index; BD, bipolar disorder; df, degree of freedom; IQR, interquartile range; MDD, major depressive disorder; SpO_2_, peripheral oxygen saturation; SD, standard deviation; χ^2^, chi-square statistic.

### 3.2 Comparison of SpO_2_ Levels Between the Two Groups

**Fig. 2.**
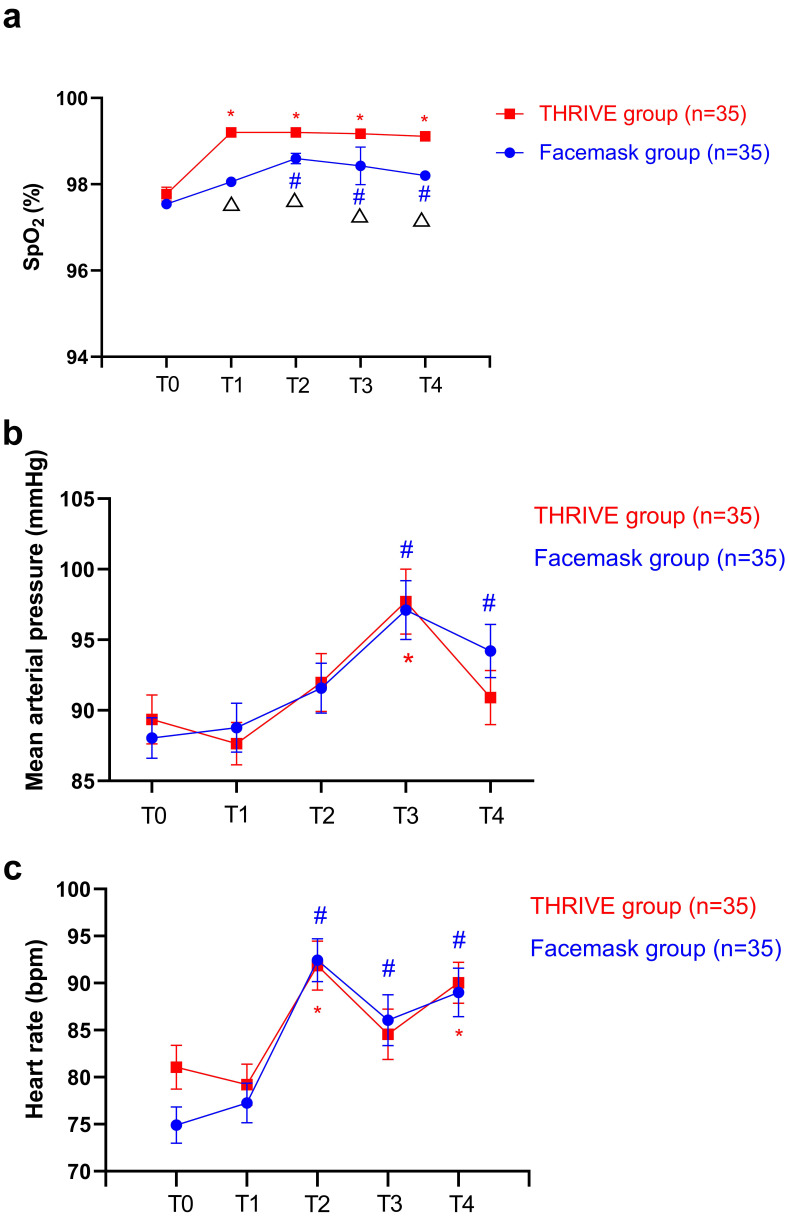
**Changes in SpO_2_ (a), mean arterial pressure (b), and heart rate (c) between the baseline and other timepoint in the THRIVE and facemask groups**.^*^Significantly different from the baseline in the THRIVE group (*p* < 0.05). ^#^Significantly different from the baseline in the facemask group (*p* < 0.05). ^△^Significant differences between the facemask and THRIVE groups at indicated times (*p* < 0.05). bpm, beat per minute.

The median lowest SpO_2_ levels in the THRIVE group were 98.0% (IQR 97.0%–99.0%), which was notably higher than those in the facemask group (97.0%; IQR 97.0%–98.0%) (*p* < 0.05), as illustrated in Table 2. Fig. 2 displays the average SpO_2_ level changes among the groups during the ECT procedure. In both groups, average SpO_2_ levels were significantly higher at T_2_, T_3_, and T_4_ compared to T_0_(all *ps* < 0.05). In the THRIVE group, average SpO_2_ levels remained significantly higher at T_1_ compared to T_0_ (*p* < 0.05). Furthermore, the THRIVE group exhibited substantially higher average SpO_2_ levels at T_1_, T_2_, T_3_, and T_4_ than the facemask group (all *ps* < 0.05). In the linear mixed model assessing average SpO_2_ levels (Table 3), the main effects of time and group were both significant (F = 18.9, *p* < 0.05 and F = 29.1, *p* < 0.05, respectively). However, the group-by-time interaction was insignificant (F = 2.2, *p* > 0.05), indicating that the average SpO_2_ levels did not show a significant difference over the ECT course for the two groups. Furthermore, during the ECT procedure, the incidence of oxygen desaturation in the THRIVE group was lower than that in the facemask group (0 versus 2.9%, *p* > 0.05).

**Table 2.** **Intraoperative data by THRIVE group versus facemask group**.

Variables		THRIVE group (n = 35)		Facemask group (n = 35)		Statistics		
		Medians	IQR	Medians	IQR	*z*	df	*p*
Lowest SpO_2_ levels during ECT procedure (%)		98.0	97.0, 99.0	97.0	97.0, 98.0	3.54	—^a^	< **0.001**
Apnea duration (minutes)		4.0	4.0, 6.0	5.0	4.0, 6.0	–1.84	—^a^	0.066
Consciousness recovery time (minutes)		10.0	9.0, 12.0	12.0	9.0, 13.0	–1.35	—^a^	0.177
Propofol dosage (mg)		110.0	100.0, 120.0	100.0	90.0, 110.0	1.48	—^a^	0.138
Succinylcholine dosage (mg)		55.0	50.0, 60.0	50.0	45.0, 55.0	1.62	—^a^	0.106
ECT parameters								
	Stimulation dosage (mC)	120.0	96.0, 168.0	120.0	96.0, 168.0	–0.13	—^a^	0.896
	Pulse frequency (Hz)	30.0	30.0, 30.0	30.0	30.0, 30.0	–0.02	—^a^	0.982
	Stimulus duration (seconds)	2.5	2.3, 3.5	2.8	2.0, 3.3	0.15	—^a^	0.881
EEG seizure duration (seconds)		25.0	22.0, 32.0	25.0	20.0, 40.0	–0.24	—^a^	0.813
Variables		THRIVE group (n = 35)		Facemask group (n = 35)		Statistics		
		N	%	N	%	$\chi^{2}$	df	*p*
SpO_2_ levels < 92%		0	0	1	2.9	—^b^	—^b^	1.000

The bolded values are *p* < 0.05. ^a^Mann–Whitney U test. ^b^Fisher’s exact test. df, degree of freedom; ECT, electroconvulsive therapy; EEG, electroencephalogram.

**Table 3.** **Comparison of SpO_2_, mean arterial pressure, and heart rate between THRIVE and facemask groups using linear mixed model analysis**.

Variables	Group-by-time interaction		Time main effect		Group main effect	
	F	*p*	F	*p*	F	*p*
SpO_2_ (%)	2.2	0.065	18.9	< **0.001**	29.1	< **0.001**
Mean arterial pressure (mm/Hg)	1.0	0.407	16.2	< **0.001**	0	0.838
Heart rate (beats per minute)	1.4	0.246	25.8	< **0.001**	1.1	0.303

The bolded values are *p* < 0.05.

### 3.3 Comparison of Anesthesia Outcomes, ECT Parameters, and EEG Seizure Duration Between the Two Study Groups

Significant differences were not observed between the THRIVE group and facemask group regarding anesthetic outcomes, including apnea duration, consciousness recovery time, propofol dosage, and succinylcholine dosage (all *ps* > 0.05; Table 2). Similarly, significant group differences were not found regarding ECT parameters and EEG seizure duration (all *ps* > 0.05; Table 2).

### 3.4 Comparison of HR and MAP at Different Time Points Between the Two Groups

Fig. 2 compares MAP and HR changes between the groups during the ECT procedure. At T_3_, both groups exhibited the most significant increase in MAP (THRIVE group versus facemask group: 97.7 ± 13.6 versus 97.1 ± 12.4 mmHg, *p* > 0.05). In both groups, MAP increased significantly only at T_3_ compared to T_0_ (*p* < 0.05). In facemask group, MAP remained significantly higher at T_4_ compared to T_0_ (*p* < 0.05). HR was significantly higher in both groups at T_2_ and T_4_ than at T_0_ (all *ps* < 0.05). Significant differences were not found between the two study groups regarding MAP and HR at each time point (all *ps* > 0.05). In the linear mixed model analysis of MAP and HR (Table 3), the main effect of time was significant (MAP: F = 16.2, *p* < 0.05; HR: F = 25.8, *p* < 0.05); however, the main effect of group were not considerable (MAP: F = 0, *p* > 0.05; HR: F = 1.1, *p* > 0.05) and there was no significant group-by-time interaction (MAP: F = 1.0, *p* > 0.05; HR: F = 1.4, *p* > 0.05).

### 3.5 Comparison of Complications Related to Airway Between Two Groups

Within 24 hours following the ECT procedure, none of the patients in the THRIVE or facemask groups reported airway-related complications, such as nose bleeds, nasal dryness, pain, or itching.

## 4. Discussion

To the best of our knowledge, this study represents the first examination of the safety and efficacy of the THRIVE technique compared to conventional facemask ventilation in Chinese patients diagnosed with MDD, BD, and schizophrenia during the ECT procedure. The main findings of this study include: (1) patients in the THRIVE group exhibited notably higher levels of the lowest SpO_2_ and average SpO_2_ throughout the ECT procedure when compared to the facemask group; (2) the incidence of oxygen desaturation in the THRIVE group was lower than that in the facemask group, while this difference did not reach significance; (3) there were no significant differences between the two groups regarding EEG seizure duration, stimulation dosage, MAP, and HR; and (4) no airway-related complications were recorded in either the THRIVE or facemask groups within 24 hours following the ECT procedure.

In this study, using the THRIVE technique under general anesthesia yielded significantly higher lowest and average SpO_2_ levels when compared to conventional facemask ventilation. However, Zhu *et al*. [27] reported that the THRIVE technique was not inferior to conventional facemask ventilation regarding the lowest and average SpO_2_ levels among ASA I-II adult patients during the ECT procedure. Similarly, Vaithialingam *et al*. [32] found no significant difference in average SpO_2_ levels between the THRIVE and facemask groups in adult patients. Several studies have identified predictors of the lowest and average SpO_2_ levels following THRIVE technique [41, 42, 43, 44, 45], and they have suggested that a lower BMI [43, 45], higher flow rates or oxygen concentrations [41, 42, 44] are associated with higher levels of the lowest SpO_2_ and average SpO_2_. Therefore, the variations of these findings could be partially attributed to the differences in BMI (patients with mean BMI ranging from 21.8 to 23.4 kg/m^2^ in previous studies [27, 32] versus 21.2 kg/m^2^ in this study), flow rate (50 L/min used in previous studies [27, 32] versus 70 L/min used in this study), and oxygen concentrations (50% oxygen applied in Zhu *et al*.’s study [27] versus 100% oxygen used in this study) across the above studies [27, 32]. It was also evident that THRIVE technology could effectively be employed in obese patients (BMI > 30 kg/m^2^) [46]. While this study found similar apnea durations in both groups, a RCT involving elderly patients demonstrated that the THRIVE technique significantly extended apnea duration compared to conventional facemask ventilation during general anesthesia [47].

Oxygen desaturation represents a potential safety concern for patients undergoing ECT procedures, leading to prolonged stays in the treatment area and incurring considerable human and material costs [12, 33]. In this study, only one patient in the facemask group experienced oxygen desaturation, while none did in the THRIVE group, aligning with the findings of prior studies [33, 34]. Jonker *et al*.’s study [33] recruited 13 patients who underwent ECT using the THRIVE technique, and none of them developed oxygen desaturation (SpO_2_ levels > 95%). Similarly, a pregnant patient successfully utilized the THRIVE technique without desaturation during ECT in her sixth month of pregnancy [34]. Given the increased risk of aspiration in pregnant patients after the first trimester, the THRIVE technique could replace conventional facemask ventilation to mitigate the associated risk [34]. However, previous studies comparing the incidence of oxygen desaturation between THRIVE and facemask groups yielded mixed findings [27, 32, 33, 34]. For instance, Vaithialingam *et al*. [32] observed that the THRIVE group had a higher incidence of oxygen desaturation compared to the facemask group (0.5% versus 0). However, Zhu *et al*. [27] found that the incidences of oxygen desaturation in the THRIVE and facemask groups were 5.3% and 6.7%, respectively. Previous studies have reported that the incidence of oxygen desaturation in patients undergoing ECT with conventional facemask ventilation ranged from 2.5% to 29.0% [8, 9, 10, 11, 12]. In summary, the above findings revealed that the THRIVE technique appeared to result in a lower incidence of oxygen desaturation during the ECT procedure compared to conventional facemask ventilation.

Following the induction of general anesthesia during apneic oxygenation, previous studies on the THRIVE technique during ECT procedures have observed a gradual rise in transcutaneous CO_2_ of 0.53 kPa/min [33]. The accumulation of CO_2_ has been linked to a shortened seizure duration and an increased seizure threshold in patients receiving ECT [48, 49]. In line with the findings of two previous studies [27, 32], this study did not identify significant differences between the THRIVE and facemask groups regarding seizure duration and stimulation dosage. Similarly, this study had no significant group differences regarding MAP and HR, consistent with earlier research [39, 47]. For example, two separate RCTs focusing on elderly patients [47] and edentulous elderly patients [39] under general anesthesia found no significant differences in HR or MAP between the THRIVE and facemask groups. Notably, Vaithialingam *et al*.’s study [32] reported a few differences in MAP and HR at selected time points; however, these differences were too minimal to have any clinical significance. The association between CO_2_ levels and several factors (e.g., flow rate, BMI, and apnea time) warrants further examination to avoid complications from elevated CO_2_ when the THRIVE technique is used as a ventilation method during the ECT procedure.

In this study, all participants undergoing ECT were followed up for 24 hours, and none of them in either THRIVE or facemask groups reported airway-related complications, such as nose bleeding, nasal dryness, pain, or itch. Similarly, numerous previous studies have also reported the safety of the THRIVE technique during ECT procedures [32, 39]. For example, Vaithialingam *et al*. [32] found that none of the patients receiving the THRIVE technique reported airway-related complications (e.g., pneumothorax, hoarseness, or nasal injury) within 24 hours following the ECT procedure. Shen *et al*. [39] reported that the THRIVE technique was utilized as safely in edentulous elderly patients as conventional facemask ventilation. The safety of the THRIVE technique might be attributed to its provision of heated and humidified oxygen [50, 51].

Several limitations should be considered in the context of this study. First, it is essential to recognize that this research was conducted at a single center and included a specific study population, limiting the generalizability of our findings. Second, blood gas analysis was not included in this study. This was not ethically justifiable for research purposes alone. Third, the sample size of this study was relatively small, which decreased the statistical power and also limited the generalizability of these findings. Furthermore, the sample size was not determined by power analysis, nor was it based on the THRIVE study in ECT. Fourth, as the participants in this study were of a healthy weight (BMI 18.5–23.9 kg/m^2^) according to the BMI classification criteria for Chinese adults [52], these findings may not apply to all patients undergoing ECT procedures. Further studies focusing on patients with overweight or obesity should be conducted. Finally, subgroup analysis based on different diagnoses was not performed in this study.

## 5. Conclusions

The THRIVE technique appeared to be more effective than conventional facemask ventilation in preserving SpO_2_ levels during ECT in patients with major mental disorders, presenting itself as a safe and effective hands-free alternative for oxygenation. However, caution is advised when applying the THRIVE technique to patients with a high BMI. Moreover, patients receiving the THRIVE technique should be given appropriate oxygenation, including an adequate flow rate and oxygen concentration.

## Availability of Data and Materials

The data used and analyzed during the current study are available from the corresponding author upon reasonable request.

## Supplementary Material

Supplementary material associated with this article can be found, in the online version, at https://doi.org/10.31083/AP39942.
